# Supplementary figures and images for: Detecting schizophrenia with 3D structural brain MRI using deep learning
Source: Sci Rep. 2023 Sep 2;13:14433. doi: 10.1038/s41598-023-41359-z (PMC10475022; doi:10.1038/s41598-023-41359-z)

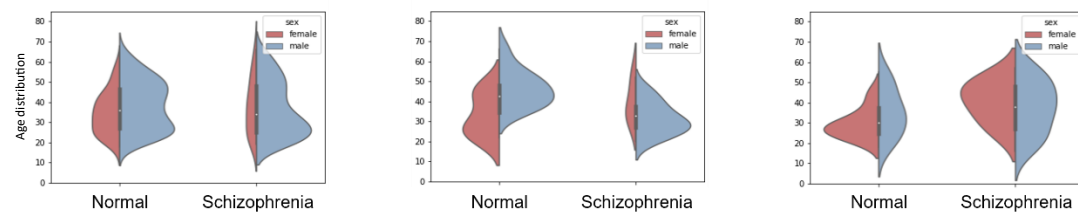

Supplementary Figure 1. The age and gender distribution in the train/validation/test dataset

Supplement: Supplementary file 1 — Supplementary Figure 1. [file 41598_2023_41359_MOESM1_ESM.pdf]
